# Determinants of COVID-19 and Influenza Vaccination Among People with Diabetes Mellitus in Primary Health Care

**DOI:** 10.3390/vaccines14070576

**Published:** 2026-06-29

**Authors:** Mariana Rodrigues Fernandes Alves Lemos, Stela de Azevedo Camtamos, Maria Eduarda Perpétuo Vilano, Silmara Nunes Andrade, Michael Jackson Oliveira de Andrade, Camila Fernanda Cunha Brandão, Ana Paula Sayuri Sato, Eliete Albano de Azevedo Guimarães, Valéria Conceição de Oliveira, Gabriela Gonçalves Amaral

**Affiliations:** 1 Department of Nursing in Collective Health, School of Nursing, University of São Paulo, São Paulo 01308-000, Brazil; marianalemos@usp.br; 2 Research Group on Infectious Disease Epidemiology with an Emphasis on Vaccination Research (EpiVac), University of São Paulo, São Paulo 01308-000, Brazil; sah@usp.br; 3 Unit Divinópolis, State University of Minas Gerais, Divinópolis 35501-170, Brazil; stela.1699999@discente.uemg.br (S.d.A.C.); maria.1656917@discente.uemg.br (M.E.P.V.); michael.andrade@uemg.br (M.J.O.d.A.); camila.brandao@uemg.br (C.F.C.B.); 4 Undergraduate Nursing Program, Federal University of São João del-Rei, Divinópolis 35501-296, Brazil; silmaranunesandrade@ufsj.edu.br (S.N.A.); elietealbano@ufsj.edu.br (E.A.d.A.G.); valeriaoliveira@ufsj.edu.br (V.C.d.O.); 5 Multiuser Center for Chronic Non-Communicable Diseases of the Midwest Region of Minas Gerais (CMDCNTs COMG), State University of Minas Gerais, Divinópolis 35501-170, Brazil; 6 Department of Epidemiology, School of Public Health, University of São Paulo, São Paulo 01246-904, Brazil

**Keywords:** primary health care, immunization programs, vaccination, diabetes mellitus, vaccination coverage, COVID-19 vaccines, influenza vaccines

## Abstract

**Background/Objectives:** People with diabetes are more susceptible to viral respiratory infections and worse clinical outcomes related to COVID-19 and influenza. Vaccination is considered an important prevention strategy. This study aimed to analyze the vaccination status against COVID-19 and influenza among people with diabetes mellitus and associated factors. **Methods:** An analytical cross-sectional study was conducted between May 2024 and May 2025 in 42 Primary Health Care Units in a municipality in Minas Gerais, Brazil. A total of 316 individuals with type 1 or type 2 diabetes mellitus participated in the study. Data were collected using a structured instrument containing socioeconomic, cultural, behavioral, and clinical variables, in addition to verification of vaccination records through physical vaccination cards and information systems. Descriptive analyses and logistic regression models were performed to estimate crude and adjusted odds ratios, with respective 95% confidence intervals. Analyses were performed using Statistical Package for the Social Sciences and Stata. **Results:** Adherence to COVID-19 vaccination was 21.5%, whereas influenza vaccination adherence reached 85.4%. In the multivariable analysis of COVID-19 vaccination status, previous influenza vaccination (OR = 7.74; 95% CI: 1.81–33.2) and alcohol consumption (OR = 2.11; 95% CI: 1.13–3.89) were positively associated with vaccination. Conversely, access to social media or other communication channels (OR = 0.47; 95% CI: 0.24–0.92) and insulin use (OR = 0.42; 95% CI: 0.21–0.84) were associated with lower odds of COVID-19 vaccination. Regarding influenza vaccination, positive associations were identified for religious affiliation (OR = 6.46; 95% CI: 1.79–23.30), previous COVID-19 vaccination (OR = 10.2; 95% CI: 2.22–47.06), and longer duration of diabetes diagnosis (OR = 3.47; 95% CI: 1.32–9.20). In contrast, alcohol consumption (OR = 0.42; 95% CI: 0.21–0.86), insulin use (OR = 0.35; 95% CI: 0.16–0.76), and absence of medical follow-up (OR = 0.34; 95% CI: 0.13–0.85) were associated with lower odds of influenza vaccination. **Conclusions:** The findings revealed a heterogeneous vaccination pattern among individuals with diabetes mellitus, in which higher influenza vaccination coverage contrasted with low adherence to COVID-19 vaccination, reflecting not only differences in the historical consolidation of immunization strategies but also contemporary dynamics related to risk perception, trust, and information circulation. The strong association with previous vaccination history suggests that vaccine adherence is part of a continuum of preventive behaviors mediated by the relationship with healthcare services and by the internalization of healthcare practices over time.

## 1. Introduction

Among chronic non-communicable diseases, diabetes mellitus stands out due to its high prevalence and the clinical, social, and economic burden it imposes on health systems worldwide [1,2,3,4,5]. The progression of the disease over recent decades has been substantial, with an estimated 537 million adults aged 20–79 years living with diabetes in 2021, and projections indicating a rise to 578 million by 2030 and 700 million by 2045 [6,7]. In Brazil, prevalence has also shown an upward trend, representing a major public health challenge, particularly given the increase in hospitalizations, complications, and healthcare costs associated with the condition [8,9,10].

In addition to cardiovascular complications, individuals with diabetes are more susceptible to infections and adverse clinical outcomes [11,12,13]. Hyperglycemia impairs immune mechanisms, increasing the risk of viral respiratory infections and progression to severe conditions, including hospitalization, intensive care unit admission, and mortality [14].

Evidence indicates that diabetes mellitus is a major risk factor for severe COVID-19 and increased mortality, particularly in cases of inadequate glycemic control [15,16,17,18,19]. Similarly, individuals with diabetes have a higher risk of hospitalization, respiratory complications, and death associated with influenza [13,16,20], making this population a priority group for preventive strategies.

In this context, vaccination represents a key preventive strategy aimed at reducing severe disease and minimizing the risk of metabolic decompensation associated with viral infections, thereby contributing to reductions in hospitalizations, metabolic complications, and mortality [21,22,23,24].

Among the vaccines recommended for this population are the influenza vaccine, administered annually, and the COVID-19 vaccine, with annual booster doses according to recommendations of the Brazilian Ministry of Health [25]. Both vaccines are available through Primary Health Care services.

Despite the free availability of these vaccines through the Brazilian Unified Health System, national studies have identified gaps in vaccination coverage and adherence to recommended schedules [22,26]. International evidence also indicates suboptimal vaccination coverage in this population [27]. Therefore, understanding vaccination status among individuals with diabetes is essential to support strategies aimed at strengthening immunization actions. Furthermore, it is assumed that socioeconomic, cultural, behavioral, and clinical determinants are associated with vaccination adherence [22,28].

Thus, this study aimed to analyze COVID-19 and influenza vaccination status and associated determinants among people with diabetes mellitus.

## 2. Materials and Methods

### 2.1. Study Design, Setting, and Period

This analytical cross-sectional study was conducted between May 2024 and May 2025 in Primary Health Care units located in a medium-sized municipality in the state of Minas Gerais, Brazil. The municipality has a territorial area of 708.115 km^2^, an estimated population of 242,505 inhabitants, and a Human Development Index of 0.764 [29]. The study followed the recommendations of the Strengthening the Reporting of Observational Studies in Epidemiology statement.

### 2.2. Population and Sample

The sampling process was based on a survey of the eligible population conducted in collaboration with the municipality’s Primary Health Care Department, using data from individuals with diabetes mellitus registered in the Electronic System of the Unified Health System Primary Health Care (e-SUS) and receiving care in Primary Health Care units, totaling 24,120 individuals (January 2024) [30].

Sample size estimation was performed using comparison of proportions, adopting a significance level of 5% and an expected prevalence of 26.1% for coverage of the 23-valent pneumococcal polysaccharide vaccine, according to a previous study [26], considering its specific recommendation for individuals with diabetes mellitus [25].

The sample was calculated based on the total number of individuals registered in the e-SUS Primary Health Care system (*n* = 24,120), using a two-stage stratified sampling design with proportional allocation according to the number of individuals with diabetes mellitus in each of the 42 Primary Health Care units in the municipality. An additional 20% was added to compensate for possible losses, resulting in a final sample of 294 participants.

### 2.3. Eligibility Criteria

Participants included individuals aged ≥18 years with a diagnosis of type 1 or type 2 diabetes mellitus, of both sexes, registered in Primary Health Care units. Cases of gestational diabetes mellitus were excluded due to the transient nature of the condition, except in situations involving a previous diagnosis of type 1 or type 2 diabetes mellitus. Individuals with visual, hearing, or cognitive impairments that could compromise adequate responses to the data collection instrument were also excluded.

### 2.4. Data Collection

Access to the study population was facilitated through collaboration with the municipality’s Primary Health Care Department and the coordination teams of the Primary Health Care units, with support from the multidisciplinary teams, especially community health workers.

Data collection was conducted individually and face-to-face through interviews carried out in previously designated areas within the Primary Health Care units or in spaces intended for collective activities, with an average duration of 30 min. To include individuals with low attendance at health units, home visits were also conducted with the support of community health workers.

All participants were informed about the voluntary nature of participation, their right to withdraw at any time without consequences, and the exclusive scientific use of the collected information. Confidentiality was ensured through the assignment of numerical codes, and participation was formalized after reading and signing the informed consent form.

### 2.5. Data Collection Instrument and Study Variables

Data were collected using an electronic form developed through the Google Forms platform, based on a structured questionnaire designed by the research team according to the study objectives and relevant literature [30]. The questionnaire underwent content validation through consensus among researchers and experts in vaccination and public health and was subsequently pilot-tested with a separate sample (*n* = 15) to assess question comprehension and instrument operationalization. Following the pilot study, minor semantic adjustments were made without structural or conceptual modifications.

The outcome variables, COVID-19 and influenza vaccination status, were assessed according to recommendations of the Brazilian National Immunization Program [25,31]. Complete vaccination status was defined as: (1) for COVID-19, documentation of at least one dose of any COVID-19 vaccine administered within the 12 months preceding data collection; and (2) for influenza, documentation of at least one vaccine dose administered in any of the years 2023, 2024, or 2025, corresponding to the period immediately preceding and during the study. The interval adopted for COVID-19 vaccination was based on the recommendation of an annual booster dose for individuals with comorbidities [32].

Explanatory variables included socioeconomic, cultural, behavioral, and clinical characteristics (Table 1).

Information regarding vaccination status was subsequently recorded in a separate data collection instrument based on verification of the printed vaccination card presented by participants. To complement the data and identify possible records not included in the physical document, immunization information systems were consulted following authorization from the municipal immunization coordination, using the National Health Card number and/or the Individual Taxpayer Registration number. The consultation, conducted within seven days after data collection, was performed by a researcher affiliated with the municipality’s Primary Health Care services and included the Integrated Health System (municipal proprietary system), the Citizen Electronic Health Record (e-SUS Primary Health Care), the National Immunization Program Information System (SI-PNI), and SI-PNI Web. Subsequently, a single database containing the complete vaccination history was consolidated.

Vaccination status assessment was independently performed by three researchers using an electronic Google Forms instrument, followed by comparison of the information in spreadsheets generated in Microsoft Excel^®^. Any discrepancies were discussed and resolved by consensus, with the participation of a fourth researcher.

### 2.6. Data Analysis

Descriptive analyses of the variables were performed. The normality of numerical variables was assessed using the Shapiro–Wilk test, and due to the absence of normal distribution, data were presented as median and interquartile range.

Associations between variables were investigated using logistic regression models, estimating crude and adjusted odds ratios (ORs) with corresponding 95% confidence intervals, considering COVID-19 and influenza vaccination status as outcomes. Socioeconomic and cultural, behavioral, and clinical explanatory variables were initially assessed in univariate analyses and individually entered into the models. Variables presenting *p*-values < 0.20 were subsequently included in the multivariable analysis.

Adjustment of the multivariable model was guided by a conceptual hierarchical framework of variables [34]. Initially, socioeconomic and cultural variables were included in the model with mutual adjustment. Subsequently, behavioral and clinical variables, considered dependent on the socioeconomic and cultural context, were incorporated while controlling for these characteristics. The final model retained only variables showing statistically significant associations (*p* < 0.05).

Analyses were performed using Statistical Package for the Social Sciences, version 21.0, and Stata, version 14.0.

## 3. Results

Among the 316 individuals with diabetes mellitus, low adherence to COVID-19 vaccination was observed (*n* = 68; 21.5%; 95% CI 17.1–26.3), whereas adherence to influenza vaccination was substantially higher (*n* = 270; 85.4%; 95% CI 81.3–88.9) (Table 2).

Among individuals vaccinated against COVID-19, there was a predominance of participants aged ≥70 years, females, self-identified as White, living with a partner, with children, affiliated with the Catholic religion, with no formal education or incomplete elementary education, retired, and with a family income of approximately two minimum wages (Table 2).

Similarly, among those vaccinated against influenza, most participants were aged ≥70 years, female, self-identified as White, living with a partner, with children, affiliated with a religion—predominantly Catholicism—with low educational attainment, retired, and with a family income of up to two minimum wages (Table 2).

Socioeconomic and cultural characteristics remained similar across participants regardless of vaccination status (Table 2).

In the univariate analysis of COVID-19 vaccination status, the sociodemographic and cultural variables sex, having children, number of children, religion, type of religion, and occupation met the pre-established criterion (*p* < 0.20) for inclusion in the multivariable logistic regression model. Similarly, age, religion, type of religion, and occupation met this criterion in the analysis of influenza vaccination status and were subsequently included in the multivariable model (Table 2).

Among individuals with diabetes who adhered to vaccination, both for COVID-19 and influenza, a highly similar behavioral profile was observed. Regarding informational behaviors, most participants reported having internet access, using electronic or mobile devices, and accessing social media or other communication channels. Concerning lifestyle habits, a low frequency of alcohol consumption and smoking was identified. In contrast, most participants reported maintaining healthy eating habits and were classified as active or very active in relation to physical activity level (Table 3).

With regard to access to vaccination, nearly all participants reported using the public healthcare system for vaccination services. Additionally, beyond adherence to influenza and COVID-19 vaccination, lower adherence to pneumococcal vaccination was observed (19.3%) (Table 3).

Behavioral characteristics remained similar among participants regardless of vaccination status (Table 3).

In the univariate analysis for the outcome COVID-19 vaccination status, variables related to informational behaviors (internet access, use of electronic or mobile devices, and use of social media or other communication channels), as well as alcohol consumption and previous adherence to influenza vaccination, met the pre-established criterion (*p* < 0.20) for inclusion in the multivariable logistic regression model. Similarly, for influenza vaccination, informational behavior variables, alcohol consumption, type of vaccination service used, and previous COVID-19 vaccination met this criterion and were subsequently included in the multivariable model (Table 3).

Regarding the clinical profile, a similar pattern was observed among individuals adherent to both COVID-19 and influenza vaccination. Most participants had been diagnosed with type 2 diabetes mellitus for more than 10 years, reported low insulin use, and greater use of oral antidiabetic drugs. Most participants also reported performing capillary blood glucose monitoring, generally at home, with a frequency of up to seven times per week. Follow-up within the public healthcare system predominated, with two or more medical appointments per year and additional follow-up with other healthcare professionals. Comorbidities were frequent in this group (Table 4).

Overall, the clinical profile remained similar among participants regardless of vaccination status (Table 4).

In the univariate analysis of clinical determinants for COVID-19 vaccination, insulin use and variables related to the practice, location, and frequency of capillary blood glucose monitoring met the pre-established criterion (*p* < 0.20) for inclusion in the multivariable logistic regression model. For influenza vaccination, the variables type and duration of diabetes mellitus diagnosis, insulin use, location and frequency of capillary blood glucose monitoring, as well as type of healthcare service and frequency of medical follow-up, also met this criterion and were subsequently included in the multivariable model (Table 4).

In the multiple logistic regression model for COVID-19 vaccination status, individuals with diabetes mellitus who had a previous influenza vaccination record were approximately seven times more likely to be vaccinated against COVID-19 (7.74; 95% CI 1.81–33.2). Alcohol consumption was also positively associated with vaccination, increasing the likelihood of vaccine adherence by approximately twofold (2.11; 95% CI 1.13–3.89). Conversely, access to social media or other communication channels and insulin use were associated with reductions of 53.0% (0.47; 95% CI 0.24–0.92) and 58.0% (0.42; 95% CI 0.21–0.84), respectively, in the likelihood of COVID-19 vaccination (Table 5).

Regarding influenza vaccination status, having a religious affiliation increased the likelihood of adherence by approximately sixfold (6.46; 95% CI 1.79–23.30). Similarly, previous COVID-19 vaccination was associated with nearly nine times greater odds of influenza vaccination (10.2; 95% CI 2.22–47.06), while a duration of diabetes diagnosis longer than 10 years was associated with an approximately 2.5-fold increase in this likelihood (3.47; 95% CI 1.32–9.20). In contrast, alcohol consumption was associated with a 58.0% reduction in the likelihood of vaccination adherence (0.42; 95% CI 0.21–0.86). Likewise, insulin use and absence of medical follow-up were associated with 65.0% (0.35; 95% CI 0.16–0.76) and 66.0% (0.34; 95% CI 0.13–0.85) lower odds, respectively, of influenza vaccination (Table 5).

## 4. Discussion

This study analyzed COVID-19 and influenza vaccination status among individuals with diabetes mellitus, revealing low adherence to COVID-19 vaccination and higher adherence to influenza vaccination. The findings indicate that behavioral, clinical, socioeconomic, and cultural determinants are differently associated with each outcome.

Overall, adherence to one vaccine was strongly associated with previous vaccination history, suggesting the existence of a consistent preventive behavior pattern over time. In addition, characteristics such as alcohol consumption, duration of disease diagnosis, and religious affiliation showed relevant associations, although in different directions depending on the vaccine analyzed. Conversely, variables such as access to communication channels, insulin use, and absence of medical follow-up were associated with lower vaccination adherence, indicating possible barriers related to healthcare, access, and quality of information. These findings reinforce the complexity of vaccination determinants among individuals with diabetes mellitus, involving individual, healthcare-related, and contextual factors.

Higher adherence to influenza vaccination compared with COVID-19 vaccination may partly be explained by the historical consolidation of influenza vaccination campaigns in Brazil and across the Americas, which have been widely incorporated into healthcare routines and population culture [35,36]. In contrast, lower adherence to COVID-19 vaccination may reflect the recent context of its implementation, marked by uncertainty, misinformation, and possible vaccine hesitancy [37,38,39,40].

This scenario of low COVID-19 vaccination coverage is consistent with studies conducted in different settings, such as China (25.2%) [41], Sudan (31.0%) [42], and Saudi Arabia (34.7%) [43]. In contrast, studies conducted in China (87.7%) [15] and Piauí, Brazil (99.7%) [22], demonstrated higher adherence to COVID-19 vaccination, particularly during the early stages of the pandemic. During that period, high-risk perception and broad exposure to the disease favored vaccine uptake [44]. As epidemiological control advanced, reduced risk perception may have contributed to declining vaccination adherence over time [45].

The association between influenza and COVID-19 vaccination adherence reinforces the hypothesis of consistent vaccination behavior among individuals with diabetes mellitus [27,46]. Integration and simultaneous implementation of vaccination campaigns may expand opportunities for access and reinforce preventive guidance [47]. Individuals with a history of vaccination tend to maintain preventive health practices over time, which may reflect stronger bonds with healthcare services, greater risk perception, and higher confidence in vaccines [48,49]. These findings reinforce the importance of strategies aimed at promoting continuity of care and strengthening a culture of prevention, especially among individuals with chronic conditions.

The association between alcohol consumption and vaccination status, although in different directions depending on the vaccine analyzed, highlights the complexity of behavioral determinants. Similar findings have been reported in the literature, although results remain inconsistent across populations and vaccine types. A systematic review including more than 5.5 million individuals found that substance use, including alcohol consumption, was associated with lower vaccine acceptance for several vaccines [50]. In the present study, alcohol consumption was associated with lower adherence to influenza vaccination, a finding that is consistent with a study conducted in Quebec, which reported higher influenza vaccination uptake among adults with chronic conditions who reported lower alcohol consumption [51]. In contrast, a study conducted among adults in the United States found that abstinence from alcohol was associated with a lower likelihood of receiving the herpes zoster vaccine [52]. Together, these findings suggest that the relationship between alcohol consumption and vaccination behavior may vary according to the vaccine analyzed, social context, health status, and characteristics of the target population.

Alcohol consumption may interfere with T- and B-cell function, compromising immune responses to vaccines [53]. However, this relationship is multifaceted and may involve behavioral and contextual determinants, making it impossible to attribute the findings exclusively to biological mechanisms. Thus, alcohol consumption may act as an indirect marker of other determinants and should be interpreted cautiously and further investigated in future studies.

Regarding clinical determinants, a longer duration of diabetes mellitus diagnosis was positively associated with influenza vaccination adherence. Similar findings have been reported in studies conducted in Singapore [54] and Spain [55], which showed that greater utilization of healthcare services, including more frequent medical visits, screening for diabetes-related complications, and previous vaccination experiences, was associated with higher vaccination uptake among individuals with chronic conditions. These findings suggest that prolonged disease duration may increase opportunities for contact with healthcare professionals and reinforce preventive health behaviors. Therefore, individuals living longer with diabetes may be more exposed to vaccination recommendations and more aware of the risks associated with infectious diseases and their complications [24,56,57]. Conversely, insulin use was associated with lower adherence, which may indicate greater clinical complexity, therapeutic burden, or difficulties related to healthcare access and organization of care [58]. These findings point to the need for tailored approaches for individuals with different clinical profiles.

Lower vaccination adherence among individuals without medical follow-up reinforces the central role of healthcare services in promoting vaccination. Consistent with our findings, studies conducted in different settings have shown that greater utilization of healthcare services is associated with higher vaccination uptake among people with diabetes mellitus. In a national survey conducted in Spain, infrequent contact with healthcare services was identified as one of the main barriers to vaccination [55]. Similarly, a study conducted in Primary Health Care in Brazil found that one of the most important reasons for incomplete vaccination schedules was the lack of guidance from healthcare professionals regarding the importance of vaccination [22].

The influence of healthcare professionals on vaccination decisions has also been widely documented. In a Spanish cohort of individuals with type 2 diabetes, approximately 90% of vaccinated participants reported accepting vaccination based on their physician’s recommendation, usually due to age or the presence of a chronic condition [59]. Similarly, studies conducted among people with diabetes in Saudi Arabia identified recommendations from family physicians and specialists as significant factors influencing vaccine uptake [60]. These findings are further supported by a scoping review that identified healthcare professionals’ recommendations as one of the most consistent positive determinants of vaccination adherence in this population [24].

The relationship with healthcare professionals facilitates access to vaccination, provides qualified guidance, and helps clarify doubts [48,49]. However, evidence also suggests that healthcare providers may face barriers to promoting vaccination, including insufficient training in adult immunization, limited consultation time, inadequate educational materials, and difficulties in identifying eligible individuals [61]. In this context, training healthcare professionals in health education is essential for them to act as reliable sources of information and support informed decision-making [62]. Literature reviews have highlighted that strengthening vaccination-related content in professional education, as well as developing communication skills to address vaccine hesitancy, are key strategies for improving vaccination coverage among people with diabetes [24,63].

The association between access to social media and other communication channels and lower adherence to COVID-19 vaccination suggests that the availability of information alone does not guarantee its quality or effectiveness. Similar concerns have been reported in the literature. A review investigating COVID-19 vaccination among individuals with diabetes identified limited knowledge about vaccines, concerns regarding their safety and effectiveness, and fear of adverse events as the main reasons for vaccine refusal [57]. The same review highlighted that misinformation disseminated through traditional media and social networks contributes to distrust toward vaccination in this population [57]. Furthermore, studies have shown that exposure to fake news is associated with lower vaccination rates and reduced intention to vaccinate [64,65]. Evidence from vaccine communication research also indicates that digital communication channels may both facilitate access to health-related content and amplify perceived barriers when the messages conveyed are redundant, conflicting, or inaccurate [66,67]. Taken together, these findings reinforce the impact of misinformation, particularly in digital environments [37,38,39,40], and suggest that vaccination adherence depends not only on access to information but also on public trust, the quality and consistency of health communication strategies, and the sociopolitical context in which vaccination campaigns are implemented.

Socioeconomic and cultural determinants also played a relevant role in vaccination adherence. Religious affiliation was positively associated with influenza vaccination. Similar findings have been attributed to the social support, trust, and sense of community often fostered by religious participation, which may facilitate the dissemination of health information and encourage preventive health behaviors [68,69]. However, evidence regarding the relationship between religion and vaccination remains heterogeneous. A population-based study conducted in Germany found that individuals with certain religious affiliations were less likely to receive influenza vaccination than those without a religious affiliation. The authors suggested that cultural differences, specific beliefs, and distinct forms of religious engagement may influence vaccination behavior [70]. Likewise, a recent scoping review concluded that religiosity may be associated with greater vaccine acceptance in some contexts, while in others it may contribute to vaccine hesitancy or refusal, depending on prevailing beliefs and the influence of religious leaders [53]. These findings suggest that the influence of religion on vaccination behavior is highly context-dependent and may vary according to cultural and social factors.

From a public health perspective, the findings of this study have important implications for the care of individuals with diabetes mellitus. The discrepancy between higher adherence to influenza vaccination and lower adherence to COVID-19 vaccination highlights the need for differentiated communication and engagement strategies, with emphasis on addressing vaccine hesitancy and misinformation. The strong association between previous vaccination history and current adherence suggests that interventions aimed at promoting a culture of prevention may produce long-lasting effects. Furthermore, integrating vaccination into routine care for chronic conditions, especially within Primary Health Care, appears essential. The need for actions directed toward subgroups more vulnerable to non-adherence should also be emphasized, considering sociocultural and behavioral aspects to promote greater equity in vaccination coverage.

As a contribution of social relevance, this study enabled the provision of individualized guidance to participants regarding the importance of vaccination for individuals with diabetes mellitus and access to recommended vaccines. Additionally, vaccination records were reviewed, transcribed, and registered in the Citizen Electronic Health Record, enabling the creation of a digital copy of vaccination cards. This strategy supports longitudinal follow-up, issuance of duplicate records, and improvement of care quality, while also contributing to the prevention of unnecessary revaccination.

This study has limitations that should be considered. The cross-sectional design does not allow causal relationships to be established between the analyzed variables and vaccination status. In addition, the use of self-reported information may be subject to recall and social desirability biases. Nevertheless, the results provide relevant contributions to understanding vaccination determinants among individuals with diabetes mellitus.

## 5. Conclusions

The findings revealed a heterogeneous vaccination pattern among individuals with diabetes mellitus, in which higher influenza vaccination coverage contrasted with low adherence to COVID-19 vaccination, reflecting not only differences in the historical consolidation of immunization strategies but also contemporary dynamics related to risk perception, trust, and information circulation. The strong association with previous vaccination history suggests that vaccine adherence is part of a continuum of preventive behaviors mediated by the relationship with healthcare services and by the internalization of healthcare practices over time.

In the context of Primary Health Care, the findings highlight the need for specific strategies to increase COVID-19 vaccination adherence, particularly among individuals with chronic conditions, as well as interventions sensitive to the social, cultural, and clinical inequalities that influence vaccination. Furthermore, the results emphasize the relevance of integrating immunization actions into routine chronic care, with emphasis on strengthening health education practices and building confidence in vaccines.

For policymakers and health managers, these findings reinforce the importance of developing targeted vaccination strategies for individuals with diabetes mellitus, including active outreach initiatives, routine assessment of vaccination status during healthcare encounters, and educational campaigns designed to address vaccine hesitancy and misinformation. Investments in interoperable immunization information systems and continuing education for healthcare professionals may further contribute to improving vaccination coverage in this high-risk population. In this regard, the present findings may support the design of evidence-based interventions aimed at increasing vaccine uptake through the integration of immunization services into chronic disease management and the reduction in social, cultural, and informational barriers to vaccination.

Thus, this study contributes to advancing the understanding of determinants of vaccination status among individuals with diabetes mellitus and provides evidence to support the development of more integrated, equitable, and responsive policies and practices within the Brazilian Unified Health System.

## Figures and Tables

**Table 1:** Description of outcome and explanatory variables. Divinópolis, Minas Gerais, Brazil, 2025.

Variables	Description
Outcome variables	COVID-19 and influenza vaccination status
Socioeconomic and cultural variables	Age; sex; race/skin color; marital status; history of children; number of children; religious affiliation; type of religion; educational level; current occupation; family income ^a^
Behavioral variables	Internet access; use of electronic/mobile devices; access to social media or other communication channels; alcohol consumption; smoking; healthy eating habits; illicit drug use; physical activity level ^b^; service used for vaccination; pneumococcal vaccination record ^c^
Clinical variables	Type of diabetes mellitus; duration of diagnosis; insulin use; use of oral antidiabetic drugs; capillary blood glucose monitoring; location of capillary blood glucose monitoring; frequency of capillary blood glucose monitoring; comorbidities; healthcare service used for medical follow-up (general practitioner/endocrinologist); frequency of medical follow-up (general practitioner/endocrinologist); follow-up with another healthcare professional

Notes: ^a^ Family income was categorized according to the Brazilian minimum wage in force in 2024 (BRL 1412.00). ^b^ Physical activity level was assessed using the International Physical Activity Questionnaire, a validated and widely used instrument that considers activities performed during the week preceding the interview [33]. ^c^ For pneumococcal vaccines, the record of one or more doses of pneumococcal vaccines was considered, including the 13-valent pneumococcal conjugate vaccine, 15-valent pneumococcal conjugate vaccine, 20-valent pneumococcal conjugate vaccine, and 23-valent pneumococcal polysaccharide vaccine [25].

**Table 2:** Descriptive and univariate analysis of COVID-19 and influenza vaccination status according to socioeconomic and cultural variables among individuals with diabetes mellitus. Divinópolis, Minas Gerais, Brazil, 2025 (*n* = 316).

Variables	Sample (*n* = 316)	COVID-19 Vaccination Schedule (*n* = 68; 21.5%)	OR (95% CI)	*p*-Value	Influenza Vaccination Schedule (*n* = 270; 85.4%)	OR (95% CI)	*p*-Value
	*n* (%)	*n* (%)			*n* (%)		
Age, years [median: 67 (60–74)]							
20–59	74 (23.4)	13 (17.6)	reference	-	56 (75.7)	reference	-
60–69	106 (33.6)	21 (19.8)	1.15 (0.53–2.49)	0.705	91 (85.9)	1.95 (0.91–4.17)	0.086
≥70	136 (43.0)	34 (25.00)	1.56 (0.76–3.19)	0.219	123 (90.4)	3.04 (1.39–6.63)	0.005
Sex							
Male	66 (20.9)	48 (19.2)	0.54 (0.29–1.00)	0.05	216 (86.4)	1.41 (0.68–2.90)	0.350
Female	250 (79.1)	20 (30.3)	reference	-	54 (81.8)	reference	-
Race/skin color							
White	167 (52.8)	35 (21.0)	reference	-	142 (85.0)	reference	-
Non-white	149 (47.2)	33 (22.1)	1.07 (0.62–1.83)	0.797	128 (85.9)	1.07 (0.57–2.00)	0.826
Marital status							
With partner	170 (53.8)	37 (21.8)	reference	-	143 (84.1)	reference	-
Without partner	146 (46.2)	31 (21.2)	0.96 (0.56–1.66)	0.909	127 (87.0)	1.26 (0.66–2.37)	0.472
Children							
Yes	271 (85.8)	55 (20.2)	0.62 (0.30–1.27)	0.197	232 (85.6)	1.09 (0.45–2.62)	0.838
No	45 (14.2)	13 (29.5)	reference	-	38 (85.6)	reference	-
Number of children [median: 2 (1–3)]							
0	45 (14.2)	13 (28.9)	reference	-	38 (84.4)	reference	-
1–2	115 (36.4)	25 (21.7)	0.68 (0.31–1.49)	0.341	100 (87.0)	1.22 (0.46–3.24)	0.679
≥3	156 (49.4)	30 (19.2)	0.58 (0.27–1.25)	0.167	132 (84.6)	1.01 (0.40–2.53)	0.978
Religion							
Yes	306 (96.8)	64 (20.9)	0.39 (0.10–4.44)	0.162	265 (86.6)	6.46 (1.79–23.30)	0.004
No	10 (3.2)	4 (40.0)	reference	-	5 (50.0)	reference	-
Religious affiliation							
Catholic	260 (82.3)	52 (20.0)	reference	-	231 (88.9)	reference	-
Evangelical	31 (9.8)	7 (22.6)	1.16 (0.47–2.85)	0.736	23 (74.2)	0.36 (0.14–0.88)	0.025
Other	15 (4.7)	5 (33.3)	2.00 (0.65–6.10)	0.223	11 (73.3)	0.34 (0.10–1.15)	0.084
No religion	10 (3.2)	4 (40.0)	2.66 (0.72–9.79)	0.140	5 (50.0)	0.12 (0.03–0.45)	0.002
Education level							
No formal education/incomplete primary education	176 (55.7)	34 (19.3)	0.71 (0.24–2.11)	0.548	154 (87.5)	0.77 (0.16–3.58)	0.747
Complete primary education	61 (19.3)	17 (27.9)	1.15 (0.36–3.68)	0.802	48 (78.7)	0.41 (0.08–2.00)	0.270
Complete secondary education	59 (18.7)	12 (20.3)	0.76 (0.23–2.52)	0.662	50 (84.8)	0.61 (0.12–3.13)	0.560
Complete higher education	20 (6.3)	5 (25.0)	reference	-	18 (90.0)	reference	-
Current occupational status							
Employed	39 (12.3)	5 (12.8)	reference	-	27 (69.2)	reference	-
Unemployed	58 (18.4)	11 (19.0)	1.59 (0.50–5.00)	0.427	50 (86.2)	2.77 (1.01–7.62)	0.047
Retired	219 (69.3)	52 (23.7)	2.11 (0.78–5.69)	0.137	193 (88.1)	3.29 (1.49–7.29)	0.003
Household income. minimum wages [median: 2 (1–3)]							
≤1	83 (26.3)	14 (16.9)	0.75 (0.35–1.60)	0.458	70 (84.3)	0.86 (0.37–1.98)	0.731
2	139 (44.0)	34 (24.5)	1.19 (0.63–2.24)	0.572	119 (85.6)	0.95 (0.44–2.02)	0.904
≥3	94 (29.7)	20 (21.3)	reference	-	81 (86.2)	reference	-

**Table 3:** Descriptive and univariate analysis of COVID-19 and influenza vaccination status according to behavioral variables among individuals with diabetes mellitus. Divinópolis, Minas Gerais, Brazil, 2025 (*n* = 316).

Variables	Sample (*n* = 316)	COVID-19 Vaccination Schedule (*n* = 68; 21.5%)	OR (95% CI)	*p*-Value	Influenza Vaccination Schedule (*n* = 270; 85.4%)	OR (95% CI)	*p*-Value
	*n* (%)	*n* (%)			*n* (%)		
Internet access							
Yes	254 (80.4)	51 (20.0)	0.66 (0.35–1.25)	0.209	214 (84.3)	0.57 (0.23–1.41)	0.229
No	62 (19.6)	17 (27.4)	reference	-	56 (90.3)	reference	-
Use of electronic/mobile devices							
Yes	254 (80.4)	56 (22.0)	1.17 (0.58–2.36)	0.644	216 (85.0)	0.84 (0.37–1.90)	0.681
No	62 (19.6)	12 (19.3)	reference	-	54 (87.10)	reference	-
Acess to social media or other communication platforms							
Yes	256 (81.0)	49 (19.1)	0.51 (0.27–0.95)	0.036	216 (84.4)	0.60 (0.24–1.48)	0.270
No	60 (19.0)	19 (31.7)	reference	-	54 (90.0)	reference	-
Alcohol consumption							
Yes	82 (25.9)	23 (28.0)	1.63 (0.91–2.92)	0.096	64 (78.1)	0.48 (0.25–0.93)	0.030
No	234 (74.1)	45 (19.3)	reference	-	206 (88.0)	reference	-
Smoking							
Yes	29 (9.2)	7 (24.1)	1.17 (0.48–2.88)	0.719	25 (86.2)	1.07 (0.35–3.23)	0.903
No	287 (90.8)	61 (21.2)	reference	-	245 (85.4)	reference	-
Healthy diet							
Yes	266 (84.2)	60 (22.6)	1.52 (0.68–3.43)	0.303	230 (86.5)	1.59 (0.73–3.47)	0.237
No	50 (15.8)	8 (16.0)	reference	-	40 (80.0)	reference	-
Illicit drug use							
Yes	1 (0.3)	0 (0.00)			1 (100.0)		
No	315 (99.7)	68 (21.6)			269 (85.4)		
Physical activity level							
Active/very active	157 (49.7)	35 (22.3)	reference	-	131 (83.4)	reference	-
Irregularly active	87 (27.5)	20 (23.0)	1.04 (0.55–1.94)	0.901	76 (87.4)	1.37 (0.64–2.93)	0.415
Sedentary	72 (22.8)	13 (18.1)	0.76 (0.37–1.55)	0.378	63 (87.5)	1.38 (0.61–3.13)	0.429
Type of vaccination service used							
Public healthcare system	309 (97.8)	68 (22.0)			267 (86.4)	reference	-
Public and private healthcare systems	4 (1.3)	0 (0.00)			2 (50.0)	0.15 (0.02–1.14)	0.068
Does not currently receive vaccination	3 (0.9)	0 (0.00)			1 (33.3)	0.07 (0.00–0.88)	0.040
Influenza vaccination status							
Yes	270 (85.4)	66 (24.4)	7.11 (1.67–30.16)	0.008			
No	46 (14.6)	2 (4.4)	reference	-			
COVID-19 vaccination status							
Yes	68 (11.5)				66 (97.1)	7.11 (1.68–30.16)	0.008
No	248 (78.5)				204 (82.3)	reference	-
Pneumococcal vaccination status							
Yes	61 (19.3)	14 (22.9)	1.10 (0.56–2.16)	0.762	55 (90.2)	1.70 (0.68–4.22)	0.249
No	255 (80.7)	54 (21.2)	reference	-	215 (84.3)	reference	-

**Table 4:** Descriptive and univariate analysis of COVID-19 and influenza vaccination status according to clinical variables among individuals with diabetes mellitus. Divinópolis, Minas Gerais, Brazil, 2025 (*n* = 316).

Variables	Sample (*n* = 316)	COVID-19 Vaccination Schedule (*n* = 68; 21.5%)	OR (95% CI)	*p*-Value	Influenza Vaccination Schedule (*n* = 270; 85.4%)	OR (95% CI)	*p*-Value
	*n* (%)	*n* (%)			*n* (%)		
Type of diabetes mellitus							
Type 1	26 (8.2)	7 (15.4)	reference	-	20 (76.9)	reference	-
Type 2	290 (91.8)	64 (22.01)	1.55 (0.51–4.68)	0.430	250 (86.2)	1.87 (0.70–4.95)	0.205
Duration of diabetes diagnosis, years [median: 10 (6–15)]							
≤5	74 (23.4)	17 (23.0)	reference	-	59 (79.7)	reference	-
6–10	114 (36.1)	22 (19.3)	0.80 (0.39–1.63)	0.544	94 (82.5)	1.19 (0.56–2.51)	0.639
>10	128 (40.5)	29 (22.7)	0.98 (0.49–1.94)	0.959	117 (91.4)	2.70 (1.16–6.25)	0.020
Insulin use							
Yes	102 (32.3)	13 (12.8)	0.42 (0.21–0.81)	0.010	82 (80.4)	0.56 (0.29–1.07)	0.081
No	214 (67.7)	55 (25.7)	reference	-	188 (87.9)	reference	-
Use of oral antidiabetic drugs							
Yes	285 (90.2)	64 (22.5)	1.95 (0.65–5.79)	0.227	245 (86.0)	1.47 (0.56–3.80)	0.428
No	31 (9.8)	4 (12.9)	reference	-	25 (80.7)	reference	-
Self-monitoring of blood glucose							
Yes	209 (66.1)	39 (18.7)	0.61 (0.35–1.07)	0.085	175 (83.7)	0.65 (0.32–1.31)	0.231
No	107 (33.9)	29 (27.1)	reference	-	95 (88.8)	reference	-
Location of blood glucose monitoring							
No monitoring	107 (33.9)	29 (27.1)	reference	-	95 (89.6)	reference	-
Home	149 (47.2)	28 (18.8)	0.62 (0.34–1.12)	0.117	123 (82.6)	0.54 (0.25–1.16)	0.118
Primary Health Care unit	49 (15.5)	7 (14.3)	0.44 (0.18–1.10)	0.083	41 (82.0)	0.52 (0.20–1.36)	0.189
Both (Primary Health Care unit and home)	11 (3.5)	4 (36.4)	1.53 (0.41–5.64)	0.517	11 (100.0)		
Frequency of blood glucose monitoring, times per week							
≤7	234 (74.1)	58 (24.8)	reference	-	204 (87.2)	reference	-
>7	82 (25.9)	10 (12.2)	0.42 (0.20–0.87)	0.019	66 (80.5)	0.60 (0.31–1.18)	0.142
Comorbidities							
Yes	224 (70.9)	49 (21.9)	1.07 (0.59–1.95)	0.810	193 (86.2)	1.21 (0.62–2.37)	0.573
No	92 (29.1)	19 (20.7)	reference	-	77 (83.7)	reference	-
Healthcare service for medical follow-up (general practitioner/endocrinologist)							
Public healthcare system	210 (66.5)	49 (23.3)	reference	-	184 (87.6)	reference	-
Private healthcare system	33 (10.4)	6 (18.2)	0.73 (0.28–1.87)	0.512	27 (81.8)	0.63 (0.23–1.68)	0.363
Both	34 (10.8)	6 (17.7)	0.70 (0.27–1.79)	0.463	31 (91.2)	1.46 (0.41–5.11)	0.554
No follow-up	39 (12.3)	7 (18.0)	0.71 (0.29–1.72)	0.461	28 (71.8)	0.35 (0.16–0.80)	0.013
Frequency of medical follow-up (general practitioner/endocrinologist), visits per year							
1	32 (10.1)	8 (25.0)	1.20 (0.51–2.84)	0.666	29 (90.6)	1.45 (0.41–5.04)	0.557
≥2	245 (77.5)	53 (21.6)	reference	-	213 (86.9)	reference	-
No follow-up	39 (12.3)	7 (18.0)	0.79 (0.33–1.89)	0.601	28 (71.8)	0.38 (0.17–0.84)	0.017
Follow-up with another health professional							
Yes	203 (64.2)	45 (22.2)	1.11 (0.63–1.96)	0.707	174 (85.7)	1.06 (0.55–2.03)	0.855
No	113 (35.8)	23 (20.3)	reference	-	96 (85.0)	reference	-

**Table 5:** Multiple logistic regression model with adjusted odds ratios for determinants of COVID-19 and influenza vaccination status among individuals with diabetes mellitus. Divinópolis, Minas Gerais, Brazil, 2025 (*n* = 316).

Variables	COVID-19 Vaccination Schedule (*n* = 68; 21.5%)	OR (95% CI)	*p*-Value	Influenza Vaccination Schedule (*n* = 270; 85.4%)	OR (95% CI)	*p*-Value
	*n* (%)			*n* (%)		
Socioeconomic and cultural variables						
Religion						
Yes				265 (86.60)	6.46 (1.79–23.30)	**0.004**
No				5 (50.00)	reference	-
Behavioral variables						
Acess to social media or other communication platforms						
Yes	49 (19.1)	0.47 (0.24–0.92)	**0.027**			
No	19 (31.7)	reference	-			
Alcohol consumption						
Yes	23 (28.0)	2.11 (1.13–3.89)	**0.017**	64 (78.05)	0.42 (0.21–0.86)	**0.017**
No	45 (19.3)			206 (88.03)	reference	-
Influenza vaccination status						
Yes	66 (24.4)	7.74 (1.81–33.2)	**0.006**			
No	2 (4.4)	reference	-			
COVID-19 vaccination status						
Yes				66 (97.1)	10.2 (2.22–47.06)	**0.003**
No				204 (82.3)	reference	-
Clinical variables						
Duration of diabetes diagnosis, years						
≤5				59 (79.73)	reference	-
6–10				94 (82.46)	1.51 (0.65–3.52)	0.332
>10				117 (91.41)	3.47 (1.32–9.20)	**0.012**
Insulin use						
Yes	13 (12.8)	0.42 (0.21–0.84)	**0.014**	82 (80.39)	0.35 (0.16–0.76)	**0.008**
No	55 (25.7)	reference	-	188 (87.85)	reference	-
Healthcare service for medical follow-up (general practitioner/endocrinologist)						
Public healthcare system				184 (87.62)	reference	-
Private healthcare system				27 (81.82)	0.53 (0.18–1.58)	0.261
Both				31 (91.18)	1.32 (0.35–4.93)	0.671
No follow-up				28 (71.79)	0.34 (0.13–0.85)	**0.022**

Note: Pseudo R^2^ (COVID-19 Vaccination Schedule): 0.1; *p* < 0.001; Pseudo R^2^ (Influenza Vaccination Schedule): 0.2; *p* < 0.001.

## Data Availability

The methodological materials and the datasets generated and/or analyzed during the present study are available in the repository at: https://doi.org/10.17632/2zwfxh8xrx.2.

## Abbreviations

The following abbreviations are used in this manuscript:

COVID-19	Coronavirus Disease 2019
OR	Odds Ratio
CI	Confidence Interval
IBGE	Brazilian Institute of Geography and Statistics
e-SUS	Electronic System of the Unified Health System Primary Health Care
BRL	Brazilian Real
SI-PNI	National Immunization Program Information System
